# Improving Boron and Molybdenum Use Efficiencies in Contrasting Cultivars of Subirrigated Greenhouse-Grown Pot Chrysanthemums

**DOI:** 10.3390/plants12122348

**Published:** 2023-06-16

**Authors:** Katherine R. Teeter-Wood, Edward J. Flaherty, Alyna J. Donetz, Gordon J. Hoover, William N. MacDonald, David J. Wolyn, Barry J. Shelp

**Affiliations:** 1 Department of Plant Agriculture, University of Guelph, Guelph, ON N1G 2W1, Canada; kteete02@uoguelph.ca (K.R.T.-W.); eflahert@uoguelph.ca (E.J.F.); jane.donetz@gmail.com (A.J.D.); ghoover@uoguelph.ca (G.J.H.); dwolyn@uoguelph.ca (D.J.W.); 2 Agricxulture Department, Niagara College Canada, Niagara-on-the-Lake, ON L0S 1J0, Canada; bmacdonald@niagaracollege.ca

**Keywords:** environmental sustainability, greenhouse floriculture, nutrient delivery, nutrient interactions, nutrient use efficiency, closed subirrigation

## Abstract

Fertilizer boron (B) and molybdenum (Mo) were provided to contrasting cultivars of subirrigated pot chrysanthemums at approximately 6–100% of current industry standards in an otherwise balanced nutrient solution during vegetative growth, and then all nutrients were removed during reproductive growth. Two experiments were conducted for each nutrient in a naturally lit greenhouse using a randomized complete block split-plot design. Boron (0.313–5.00 µmol L^−1^) or Mo (0.031–0.500 µmol L^−1^) was the main plot, and cultivar was the sub-plot. Petal quilling was observed with leaf-B of 11.3–19.4 mg kg^−1^ dry mass (DM), whereas Mo deficiency was not observed with leaf-Mo of 1.0–3.7 mg kg^−1^ DM. Optimized supplies resulted in leaf tissue levels of 48.8–72.5 mg B kg^−1^ DM and 1.9–4.8 mg Mo kg^−1^ DM. Boron uptake efficiency was more important than B utilization efficiency in sustaining plant/inflorescence growth with decreasing B supply, whereas Mo uptake and utilization efficiencies appeared to have similar importance in sustaining plant/inflorescence growth with decreasing Mo supply. This research contributes to the development of a sustainable low-input nutrient delivery strategy for floricultural operations, wherein nutrient supply is interrupted during reproductive growth and optimized during vegetative growth.

## 1. Introduction

Sufficient supplies of essential nutrients are required to sustain normal plant growth during floricultural crop production. In situations of inadequate nutrient supply, root growth generally decreases less than shoot growth, so nutrient uptake is favoured [1,2]. In addition, roots absorb fewer nutrients during reproductive growth than vegetative growth. Reproductive growth is primarily dependent on the mobilization and retranslocation of nutrients previously stored in vegetative organs. On the other hand, low nutrient supply during vegetative growth can induce high-affinity nutrient uptake transporters in the root. Thus, it has been hypothesized that nutrient use efficiency (NUE) in greenhouse floriculture can be improved by supplying moderate nutrient levels during vegetative growth and removing the entire nutrient supply at the beginning of reproductive growth [3,4].

Closed subirrigation systems have been developed to reduce operating costs and waste by containing leachate and facilitating the recycling of nutrient-rich solutions [5,6,7]. Notwithstanding these advantages, current fertilizer recommendations are still based on outdated overhead irrigation [8], so a re-evaluation of the nutrient supply for subirrigation is required. Several fertilizer formulations are available commercially for chrysanthemum production, including Peter’s Professional (Peat-Lite Neutral Cal-Mag 17-3-17, ICL Fertilizers, Dublin, OH, USA) [9], Fusion Plant-Prod (17-5-17, Master Plant-Prod, Brampton, ON, USA) [10], the Hoagland solution [11], and the modified Sonneveld solution [12]. While the macronutrient levels of these four solutions at full- or near full-strength tend to be similar, they have a broad range of micronutrient concentrations (Table S1). Previous research from the Shelp lab group confirmed that the aforementioned delivery strategy reduces the requirements for many of the essential nutrients in modern cultivars of greenhouse-grown subirrigated pot chrysanthemums by up to 94% without compromising crop/inflorescence yield and quality [3,4,13,14,15,16,17]. Boron (B) and molybdenum (Mo) are the only two essential nutrients that have not yet been studied. 

In plants, B is involved in maintaining the structural and functional integrity of cell walls and membranes, ion fluxes, cell division and elongation, and a variety of pathways related to metabolism and transport [18,19,20]. Common symptoms of B deficiency include leaf brittleness, limited meristem development and apical dominance, small leaves and short internodes, and poor structural and colour development of flowers, seeds, and fruit [21]. Petal quilling and loss of flower colour can occur in B-deficient chrysanthemums [22]. It is well established that B moves from roots to shoots through the xylem and that limited B transport occurs via the phloem, especially to young tissues, in plants that produce and transport B-sucrose complexes [23,24,25,26,27,28,29,30,31,32,33]. Foliar-applied B is also transported to other plant parts [34,35]. More B is retranslocated from the leaves of lupin plants receiving an interrupted supply compared to plants receiving adequate B, and much of the retranslocated B is cycled through the roots before being delivered to the developing inflorescence [27]. 

Molybdenum is an essential component of molybdenum cofactor (Moco), which is required for four key plant enzymes, including nitrate reductase, xanthine dehydrogenase, aldehyde oxygenase, and sulfite oxidase [36]. Molybdenum deficiency often results in leaf chlorosis and other leaf deformities, poor development of reproductive tissues, as well as altered nitrogen and sulphur metabolite profiles [37,38,39]. Transpiration drives the transport of Mo upward through the xylem, but Mo is considered to be highly remobilized during times of deficiency [2]. Early research demonstrated that Mo applied to the primary leaf of a bean plant is translocated to other plant parts, though most of it moves down to the stems and roots [40]. The translocation of Mo from root to shoot is less than that of rubidium, an analog of potassium [25]. In certain species, such as bean and sunflower, Mo preferentially accumulates in the xylem parenchyma of roots and stems, whereas, in other species such as tomato, Mo is readily translocated from root to shoot [2].

The removal of the entire nutrient supply during reproductive growth does not affect chrysanthemum yield and quality, suggesting that both B and Mo may be remobilized more during reproductive growth than vegetative growth [4,13,15,16,17,18,41]. In the present study, the delivery of B and Mo to three modern cultivars of subirrigated chrysanthemum was optimized during the vegetative stage, and nutrient utilization and uptake efficiencies were determined to improve our understanding of the mechanisms involved in sustaining plant growth with decreasing nutrient supply. 

## 2. Results

### 2.1. Growth of Two Chrysanthemum Cultivars Supplied with Moderate to Deficient Boron Levels

#### 2.1.1. Summary of Significant Effects 

**Figure 1:**
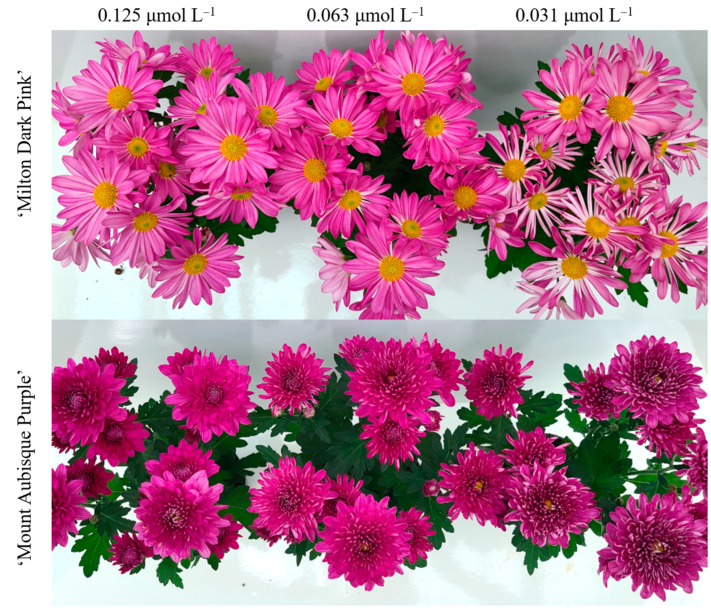
Representative inflorescences at harvest in two chrysanthemum cultivars supplied with varying levels of B prior to bud emergence (experiment 2).

Experiment 1 reduced the B supply by 75% (from 5.00 to 1.25 μmol L^−1^) without causing visual symptoms of B deficiency. Further reduction of the B supply by 88% and 94% (0.625 and 0.313 μmol L^−1^, respectively) in experiment 2 resulted in moderate and pronounced petal quilling (Figure 1), respectively, but other potential symptoms of B deficiency, such as brittleness and leaf cracking, were not observed. Treatment effects were absent for shoot height, shoot DM, bud/inflorescence DM, bloom diameter, bud/inflorescence number, and inflorescence development over time (Tables S2 and S3).

Treatment effects on nutrient levels in the diagnostic leaf at bud emergence were observed with Cu only in experiment 1 and with B only in experiment 2 (Table S4). Cultivar effects were evident for all morphological characteristics at harvest, except for shoot height in experiment 2 (Tables S2 and S3). The effects of time, treatment x cultivar, and time x cultivar on bud/inflorescence development were evident in both experiments (Table S3). Furthermore, cultivar effects were observed at many leaf nutrient levels (Table S4). Since several treatment x cultivar interactions for morphological characteristics were evident (Table S2), the cultivars are individually analyzed below.

#### 2.1.2. Morphological Characteristics

Most morphological characteristics at harvest, including shoot height, shoot (including bud/inflorescence) DM, bud/inflorescence DM, bloom diameter, and inflorescence stage, were unaffected by B treatment in experiment 1, regardless of the cultivar (Table 1). The only exception was a slight decrease in the bud/inflorescence number in ‘Milton Dark Pink’ with decreasing B. In experiment 2, the morphological characteristics of ‘Williamsburg Purple’ were all unaffected by B treatment, whereas the shoot DM and bud/inflorescence DM of ‘Milton Dark Pink’ increased slightly with decreasing B.

#### 2.1.3. Bud and Inflorescence Development

**Figure 2:**
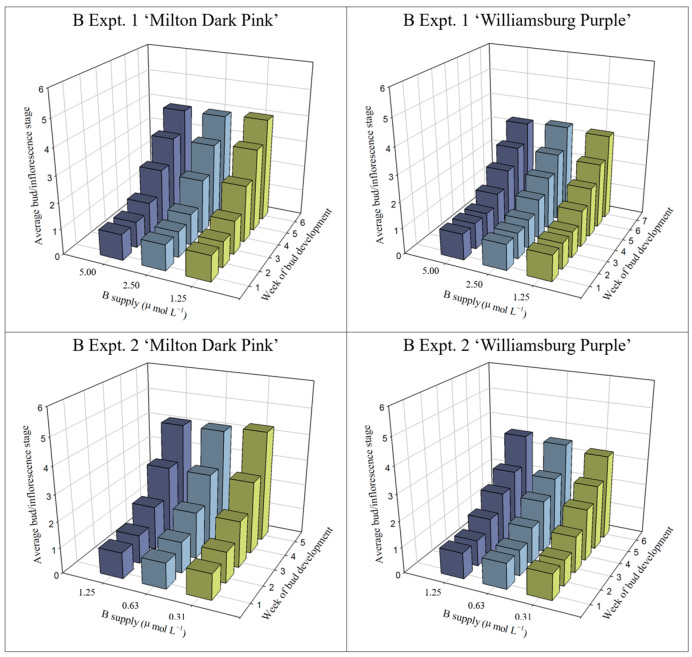
Inflorescence development of ‘Milton Dark Pink’ and ‘Williamsburg Purple’ chrysanthemums supplied with varying levels of B prior to bud emergence. Inflorescence development was measured weekly, from bud emergence (stage 1) to harvest. Stage 1: a formed bud that is completely closed; stage 2: the bloom beginning to emerge from the bud with visible petal colour; stage 3: the bloom opening with fully vertical petals but still mostly closed with sepals approximately half the petal length; stage 4: the bloom opening and mostly open; stage 5: a fully opened bloom; stage 6: a fully opened bloom in the early stages of petal-tip senescence with fully opened disk flowers. Statistical treatment of the data is shown in Table S5.

In general, the bud/inflorescence number over time from bud emergence to harvest was not affected, or slightly affected, by B treatment, regardless of the cultivar (Table S5). Except for a slight decrease in ‘Milton Dark Pink’ with decreasing B, bud/inflorescence development in experiment 1 was unaffected by B treatment (Figure 2, Table S6). In contrast, inflorescence development in experiment 2 was slightly stimulated in ‘Milton Dark Pink’ and slightly delayed in ‘Williamsburg Purple’ with decreasing B.

#### 2.1.4. Leaf Nutrient Composition at Bud Emergence

Except for a slight increase in leaf K in ‘Milton Dark Pink’ in experiment 1 and declines in leaf B (approximately 50% and 70% from 1.25 to 0.63 and 0.31 μmol L^−1^, respectively) with decreasing B supply in experiment 2, leaf nutrient composition at bud emergence was unaffected by the 16-fold range in B supply (Table 2 and Table 3). Across all cultivars and experiments, leaf B levels ranged from 11.3 to 49.0 mg kg^−1^ DM.

#### 2.1.5. Nutrient Use Efficiency

**Figure 3:**
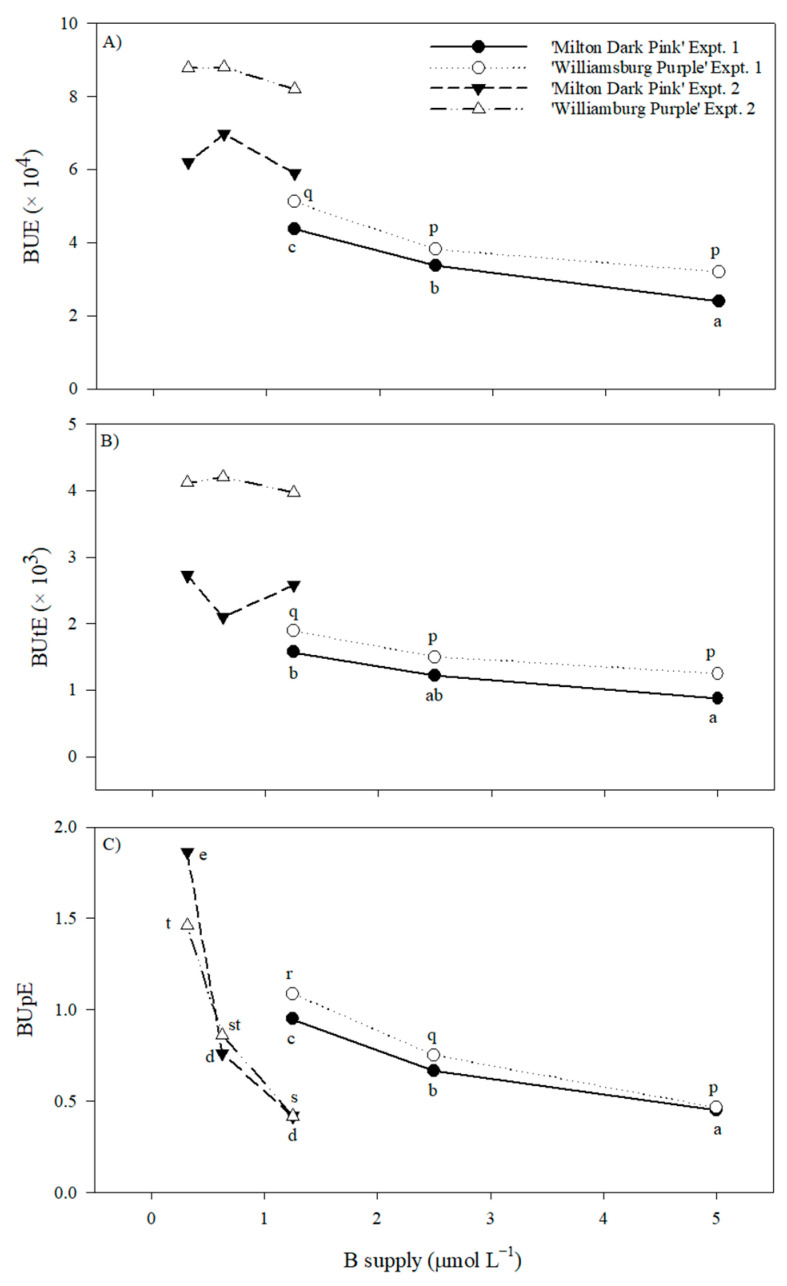
Boron use efficiency (BUE) (**A**), boron utilization efficiency (BUtE) (**B**), and boron uptake efficiency (BUpE) (**C**) of ‘Milton Dark Pink’ and ‘Williamsburg Purple’ supplied with varying levels of B prior to bud emergence (experiment 1: 5.00–1.25 μmol L^−1^) and Winter/Spring 2022 (experiment 2: 1.250–0.313 μmol L^−1^). Means (n = 4) that are significantly different (*p* ≤ 0.05) within each panel, cultivar, and experiment according to Tukey’s honest significant difference test are designated by different letters.

The accumulation of nutrients in the shoots of both cultivars at harvest was mostly unaffected by the 16-fold range in B supply (Tables S7 and S8). The sole exceptions were slight non-linear differences in Cu and Zn with ‘Milton Dark Pink’ in experiment 1 and declines in B in both cultivars in experiment 1 (approximately 23% and 45% from 5.00 to 2.50 and 1.25 μmol L^−1^, respectively). Shoot-B accumulation across all cultivars and experiments ranged from 0.46 to 2.33 mg shoot^−1^ DM. In experiment 1, B use efficiency (BUE), B utilization efficiency (BUtE), and B uptake efficiency (BUpE) increased with decreasing B supply by approximately two-fold (Figure 3). With further decreases in B supply in experiment 2, BUE and BUtE were unaffected, whereas BUpE increased by approximately four-fold. 

### 2.2. Growth of Three Chrysanthemum Cultivars Supplied with Moderate Molybdenum Levels

#### 2.2.1. Summary of Significant Effects

The full nutrient suite was provided to chrysanthemums until bud emergence only. Molybdenum supply was reduced by 75% in experiment 1 (from 0.500 to 0.125 μmol L^−1^) and by 94% in experiment 2 (from 0.125 to 0.031 μmol L^−1^) without causing visual symptoms of Mo deficiency, such as chlorotic leaves, limited reproductive structure development, and reduced development of the apical meristem (Figure S1). A treatment effect was only observed for leaf greenness in experiment 2, whereas all other harvest characteristics were unaffected (Table S9). Inflorescence development over time was unaffected by the treatment (Table S10). Of the tissue nutrients in the diagnostic leaf at bud break, treatment effects were only observed with Mo and B levels in experiment 2 (Table S11). Except for shoot height, most harvest characteristics and leaf greenness at bud break showed cultivar effects in both experiments (Table S9). The effects of time, cultivar, treatment x cultivar, and time x cultivar were also observed for inflorescence development (Table S10). Many nutrient concentrations in recently matured leaves at bud break exhibited cultivar effects as well (Table S11). Since several morphological characteristics showed a treatment x cultivar effect, the cultivars are individually analyzed below. 

#### 2.2.2. Morphological Characteristics

Reducing the Mo supply by 94% (from 0.500 to 0.031 μmol L^−1^) did not affect morphological characteristics at harvest, including shoot height, shoot DM, inflorescence/bud DM, bloom diameter, inflorescence development, and inflorescence/bud number for ‘Milton Dark Pink’, ‘Williamsburg Purple’, or ‘Mount Aubisque Purple’ (Table 4). The only exceptions were slight non-linear differences in bud/inflorescence DM and inflorescence development of ‘Milton Dark Pink’ in experiment 1 in response to decreasing Mo supply. In experiment 1, a 75% reduction in Mo supply (from 0.500 to 0.125 μmol L^−1^) resulted in a slight non-linear difference in the greenness of recently matured leaves at bud emergence in ‘Milton Dark Pink’, but not in ‘Williamsburg Purple’ (Table 4). In experiment 2, the 94% reduction in Mo supply resulted in a non-linear difference in the leaf greenness of ‘Mount Aubisque Purple’, but not in ‘Milton Dark Pink’.

#### 2.2.3. Bud and Inflorescence Development

**Figure 4:**
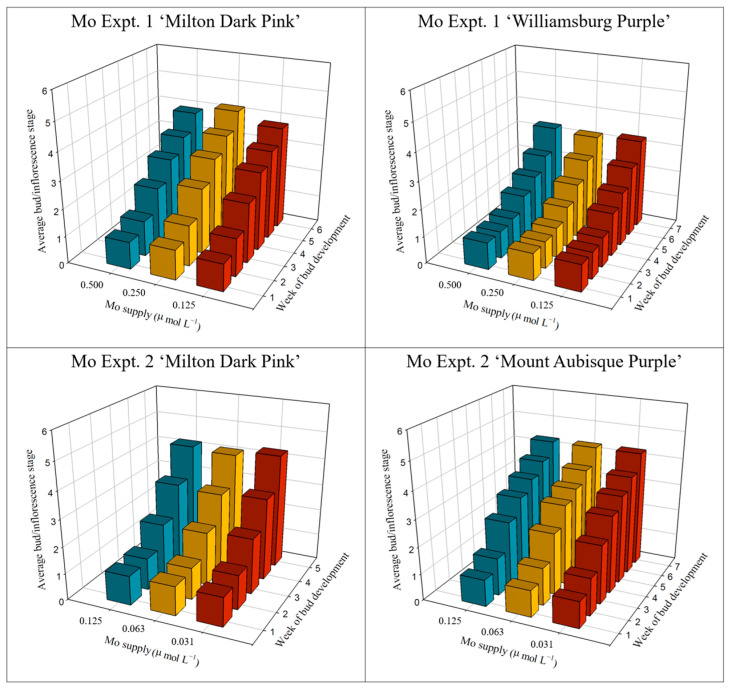
Inflorescence development of ‘Milton Dark Pink’, ‘Williamsburg Purple’, and ‘Mount Aubisque Purple’ supplied with varying levels of Mo prior to bud emergence. Inflorescence development was measured weekly, from bud emergence (stage 1) to harvest. Inflorescence stages are described in the legend of Figure 2. Statistical treatment of the data is shown in Table S13.

Mo supply did not affect the bud/inflorescence number from bud emergence to harvest in either experiment (Table S12). In addition, bud/inflorescence development was generally unaffected by Mo supply (Figure 4, Table S13). However, differences in the bud/inflorescence development were evident for ‘Milton Dark Pink’ in experiment 1, but these differences were slight and not related in a linear manner to Mo supply. 

#### 2.2.4. Leaf Nutrient Composition

Leaf nutrient composition at bud emergence was mostly unaffected by the 16-fold range in Mo supply, apart from a non-linear change in P in ‘Mount Aubisque Purple’ in experiment 2, slight decreases in B in both cultivars in experiment 2, and marked decreases in Mo (approximately 25% and 35% from 0.125 to 0.063 and 0.031 μmol L^−1^, respectively) with decreasing Mo supply in experiment 2 (Table 5 and Table 6). Leaf Mo levels across all cultivars and experiments ranged from 1.0 to 3.7 mg kg^−1^ DM. 

#### 2.2.5. Nutrient Use Efficiency

**Figure 5:**
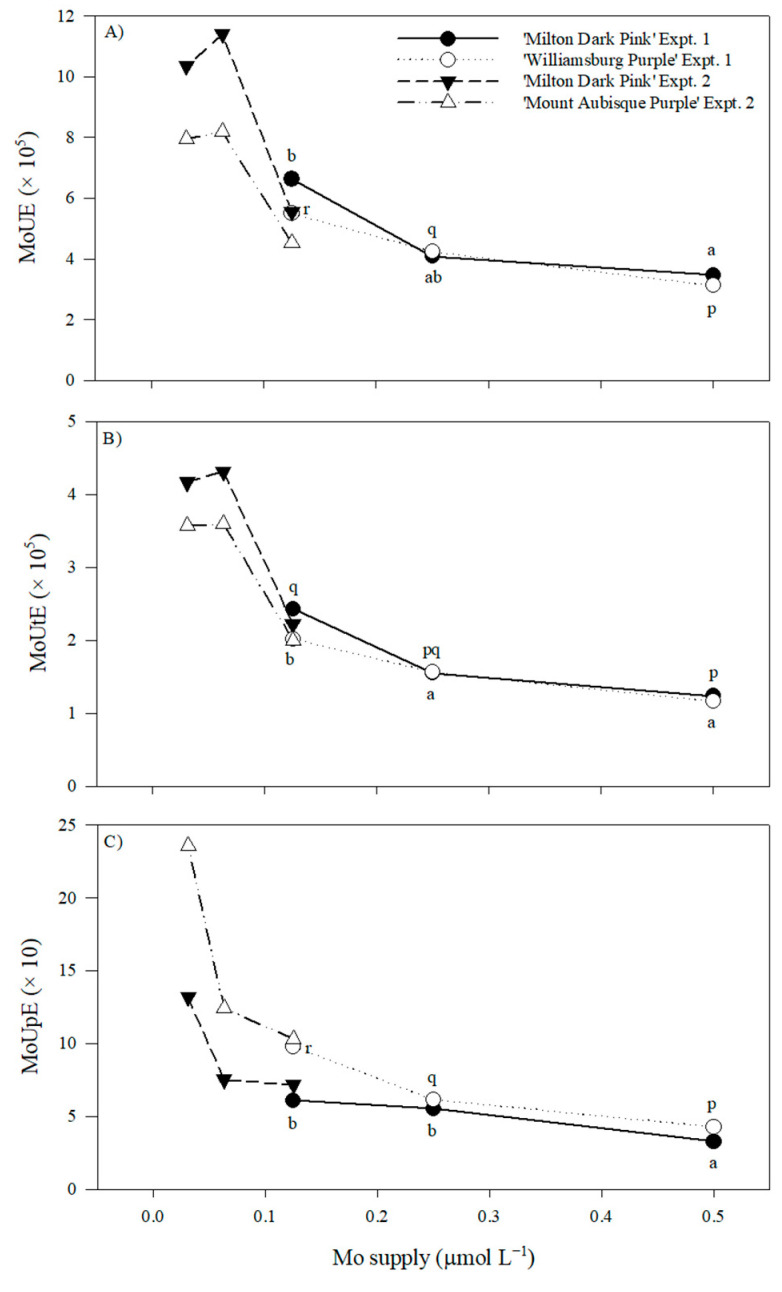
Molybdenum use efficiency (MoUE) (**A**), molybdenum utilization efficiency (MoUtE) (**B**), and molybdenum uptake efficiency (MoUpE) (**C**) of ‘Milton Dark Pink,’ ‘Williamsburg Purple,’ and ‘Mount Aubisque Purple’ supplied with varying levels of B prior to bud emergence (experiment 1: 5.00–1.25 μmol L^−1^) and Winter/Spring 2022 (experiment 2: 1.250–0.313 μmol L^−1^). Means (n = 4) that are significantly different (*p* ≤ 0.05) within each panel, cultivar, and experiment according to Tukey’s honest significant difference test are designated by different letters.

With the exception of a non-linear difference in Ca accumulation in ‘Milton Dark Pink’, the macronutrient accumulation in the shoots of both cultivars was unaffected by the 94% reduction in Mo supply (Table S14). Non-linear differences in B and Mn accumulation in ‘Milton Dark Pink’ were found in experiments 1 and 2, respectively (Table S15). Both cultivars exhibited decreases in Mo accumulation in experiment 1 (approximately 21% and 47% from 0.500 to 0.250 and 0.125 μmol L^−1^, respectively), whereas only ‘Milton Dark Pink’ showed a significant decrease in experiment 2 (approximately 45%). Across all cultivars and experiments, shoot Mo accumulation ranged from 0.05 to 0.21 mg shoot^−1^ DM. Molybdenum use efficiency (MoUE), Mo utilization efficiency (MoUtE), and Mo uptake efficiency (MoUpE) increased by approximately two-fold across the two cultivars with decreasing Mo supply in experiment 1 (Figure 5). Unfortunately, with the wide variation in the biological replicates, significant changes in the indices for Mo use efficiency were not detected with further decreases in Mo supply.

## 3. Discussion

### 3.1. Optimization of Boron and Molybdenum Fertilization

Here, greenhouse-grown chrysanthemums received up to 94% less B and Mo during vegetative growth than recommended by the industry guidelines. Boron deficiency symptoms, such as brittle leaves and petal quilling, were not observed at leaf tissue levels of 41.8–49.0 mg kg^−1^ DM, which is considered to be within the sufficiency range of 20–200 mg kg^−1^ DM established in the extension literature [42,43,44]. However, petal quilling was observed at leaf tissue levels of 11.3–19.4 mg kg^−1^ DM, which are considered below the sufficiency range in the literature. Molybdenum deficiency symptoms, such as chlorotic leaves, were not observed, regardless of the Mo supply or tissue levels, 1.0–3.7 mg kg^−1^ DM, which are above the established sufficiency range for dicotyledonous plants (0.1–1.0 mg kg^−1^ DM) [45]. Notably, when B and Mo supplies were reduced from industry standards, tissue B and Mo were 48.8–72.5 mg kg^−1^ DM and 1.9–4.8 mg kg^−1^ DM, respectively. When combined with the consistent morphological results, these findings lead to the conclusion that B and Mo use efficiencies improved approximately 8- and 32-fold, respectively, over the crop cycle, with decreasing nutrient supplies without adverse effects on plant and flower quality. These results were achieved by decreasing B and Mo supplies during vegetative growth, followed by the removal of the entire nutrient supply during reproductive growth.

The present study provided three contrasting chrysanthemum cultivars with an optimized macronutrient and micronutrient regimen (not including B and Mo) during vegetative growth across all experiments. In recently matured diagnostic leaves collected at bud emergence, the tissue levels of N (4.82–6.60% DM), P (0.54–1.02% DM), K (4.89–6.95% DM), Ca (1.02–1.71% DM), Mg (0.35–0.74% DM), Zn (21.0–53.5 mg kg^−1^ DM), Cu (2.7–7.1 mg kg^−1^ DM), Fe (70.3–119.8 mg kg^−1^ DM), and Mn (57.3–115.0 mg kg^−1^ DM) across all treatments aligned with established sufficiency levels in extension literature (4.0–6.5% DM N, 0.2–1.2% DM P, 1.0–10.0% DM K, 0.5–4.6% DM Ca, 0.1–1.5% DM Mg, 5–250 mg kg^−1^ DM Zn, 5–50 mg kg^−1^ DM Cu, 20–750 mg kg^−1^ DM Fe, and 25–375 mg kg^−1^ DM Mn) [42,43,44]. Copper could be described as low; however, Cu deficiency symptoms, such as desiccation of leaf margins and flowering suppression, were not observed [46].

The approach used here is based on an understanding of nutrient acquisition and redistribution in plants. The primary source of nutrients for the growth of young plants is the root system, but as the plant matures, previously acquired and stored nutrients become more important than root nutrient uptake, especially for fruit and flower development [1,2]. Efficient nutrient absorption early in the plant’s growth cycle and improved nutrient redistribution to reproductive structures late in the plant’s growth cycle can be induced by intentionally decreasing nutrient supply rates [1,41,47]. Consequently, fertilizer supply can be interrupted during a plant’s growth cycle when sufficient nutrients are stored in the leaves to sustain reproductive growth. Typically, nutrients are mobilized efficiently at the onset of flowering when the uptake of nutrients through the root system starts to decline [47]. This strategy can be combined with a reduction in nutrient supply to young plants, provided it is not excessive, and the nutrient uptake efficiency by roots is improved so that the plant stores the same amount of nutrients as with a much higher nutrient supply.

MacDonald et al. [13] first demonstrated that chrysanthemum plant and flower quality are unaffected by ceasing all nutrient delivery during reproductive growth. Subsequent studies have combined this practice with a reduction in specific essential nutrients in otherwise balanced solutions during vegetative growth, resulting in overall savings of 75–94% [3,4,14,16,17]. These cultivars had variable phenotypes, including biomass accumulation, bloom diameter, inflorescence type, and tissue nutrient levels; however, decreasing nutrient delivery always increased the NUE, and any minor morphological treatment effects were unnoticeable to consumers.

Luxury nutrients are commonly supplied to ornamentals grown in commercial greenhouse operations to prevent deficiency symptoms and ensure plant marketability. Although these levels are higher than necessary for maximal growth, they are not toxic. The nutrient regimens are typically based on overhead irrigation systems for outdated cultivars and are focused on N, P, and K supplies. Over the past two decades, the industry has been increasingly adopting nutrient recycling systems (i.e., closed systems), such as subirrigation and drip irrigation, to reduce waste; however, the composition of the nutrient solution is likely still based on overhead irrigation [5,6,7]. As environmental stewardship becomes increasingly important [48,49,50], there is a need to optimize fertilizer recommendations for irrigation systems and cultivars used in modern floricultural operations.

### 3.2. Mechanisms for Improved Boron and Molybdenum Use Efficiencies

Increasing the NUE for any nutrient (NtUE) as a function of its decreasing supply can result from improvements in NtUtE and/or NtUpE. Nutrient budgets were used previously to demonstrate that the primary mechanism to obtain sufficient N, P, or S for chrysanthemum growth with decreasing nutrient supply increased NtUpE [3,4,13,14]. The present study compared shoot nutrients with plant DM and nutrient supply, indicating that BUpE was more important than BUtE to sustain plant/flower growth with decreasing B supply, especially when tissue B was deficient. In contrast, MoUpE and MoUtE appeared to have similar importance in sustaining plant/flower growth with decreasing Mo supply; however, the supply and tissue levels never resulted in symptoms of Mo deficiency. This apparent discrepancy between the two elements could be related to differences in their remobilization within plants [2,23].

Boron uptake efficiency could be improved by upregulating the synthesis of plasma membrane transporters responsible for the movement of B from the soil to the shoot. In particular, this would improve the following: (i) facilitated diffusion of boric acid via uptake channels such as NIP5;1 (aquaporin protein family) in the root cap and epidermal cells from the soil to the endoderm; (ii) specific transport of boric acid/borate via BOR1/2 at the endodermis in meristematic and maturation zones; and (iii) transport of B via NIP6;1 from xylem to phloem in the stele parenchyma of shoot nodal regions [51]. Previous research showed that B limitation inhibits growth and shoot B accumulation in the atbor1-1 mutant, whereas sufficient B down-regulates AtBOR1 expression [52,53,54]. Furthermore, low-B conditions reduce expansion and B levels of young rosette leaves in the atnip6;1 mutant compared to wild-type plants, whereas old leaves are unaffected [21].

The level of cell wall-bound B in plants is relatively uniform across leaf positions and B concentrations, but the decreasing upward concentration gradient of B is related to the levels of water-soluble B (i.e., free and semi-bound forms) [31,55]. Under B deficiency, the proportion of cell wall-bound B increases in old leaves but decreases in roots, whereas the proportion in young leaves is unaffected by B supply [32]. Water-soluble B is retranslocated from fully expanded leaves, young leaves, and roots of low-B plants [31,32,56], so it probably accounts for any increase in BUtE of chrysanthemums supplied with a low input of B.

Improved MoUpE in chrysanthemums is likely associated with increased levels of a high-affinity molybdate transporter in the roots, although its subcellular localization in Arabidopsis (AtMOT1;1) is ambiguous [57,58]. Under low Mo supply, the atmot1;1 mutant accumulates less Mo in both roots and shoots than the wild type and exhibits symptoms of Mo deficiency. The rice MOT1;1 gene is mainly expressed in the roots and exhibits molybdate transport activity [59]. The osmot1;1 mutant decreases Mo translocation from roots to shoots, lowers the Mo level in grains, and enhances the sensitivity to Mo deficiency.

Different mechanisms could be associated with the improved MoUtE with decreasing Mo supply. Molybdate is stored in the vacuole but is released under Mo deficiency via the tonoplast-localized AtMOT1;2 into the cytosol, where it is incorporated into Moco [39,60]. Moco is rapidly incorporated into one of the five enzymes for which it is a prosthetic group [61]. Moreover, the levels of anthocyanin and malate in Brassica sp. and Medicago sativa L. are positively correlated with molybdate accumulation, suggesting that Mo or Moco is sequestered as organic complexes [62,63,64]. In some species, Mo preferentially accumulates in the xylem parenchyma of the roots and stems, resulting in a decreasing upward gradient [2]. In contrast, Mo is readily translocated from the roots to leaves in other species, and Mo remobilization is higher during reproductive growth than during vegetative growth [2]. Research from our laboratory reported that the entire nutrient supply, including Mo, can be removed during the reproductive growth stage without negatively affecting the production and quality of flowering chrysanthemums, suggesting that Mo is remobilized in chrysanthemums during reproductive growth [3,4,14].

An evaluation of the relative contributions of the nutrient solutions compared to the cuttings, soil mixture, and Jiffy plugs would be beneficial; however, our methods did not allow for this estimation of nutrient balance. The supply of the stable isotope ^10^B or the radioactive isotope ^99^Mo through the root system or the leaf flap feeding method could be used throughout inflorescence development to track B or Mo uptake, storage, and translocation [24,35,65]. Insight into the mechanisms responsible for B and Mo uptake and remobilization could be obtained by monitoring the expression of genes for B and Mo transporters found in roots and shoots under low, but adequate, nutrient supply to avoid the development of morphological deficiency symptoms. Improved NtUE could be influenced by root architecture, the release of root storage pools, and other factors; however, these possibilities are beyond the scope of this study.

### 3.3. Potential Interactions of Boron or Molybdenum with Other Elements

Boron influences many plant processes because it is involved in the cell wall and plasma membrane integrity [18,66]. Consequently, B deficiency causes many anatomical, physiological, and biochemical changes in plants. However, most of these probably represent secondary effects, complicated by differences in physiological age between normal and deficient tissues. It has been suggested that B plays a role in calcium metabolism in the cell wall [67,68]. Early evidence suggests that Mo interacts with iron, sulphur, and phosphorus metabolism at many levels, including (i) positive or negative effects on uptake mechanisms; (ii) requirement for iron-containing redox groups, such as iron–sulphur clusters or heme in most molybdoenzymes; (iii) the involvement of iron–sulphur cluster synthesis in Mo metabolism; and, (iv) the involvement of a specific mitochondrial ABC-type transporter in both Moco synthesis and extramitochondrial iron–sulphur proteins [36,69,70]. However, a more recent interpretation suggests that iron availability is a crucial regulatory element for plant Mo metabolism, but Mo availability is of subordinate importance for Fe metabolism [36]. Furthermore, the phosphorus uptake system may effectively bind and accumulate molybdate; however, it would appear to have a limited impact on molybdate transport under good growing conditions where the soil has adequate amounts of available phosphorus [70]. Our study provided no evidence for the interaction of B with the accumulation and remobilization of calcium when plants received moderate to deficient supplies during vegetative growth (calculations of indices for calcium use efficiency are not shown). Furthermore, there was no evidence for the interaction of Mo with the accumulation and remobilization of iron, sulphur, or phosphorus when plants received a range of adequate Mo levels during vegetative growth (calculations of indices for iron, sulphur, and phosphorus use efficiencies are not shown).

### 3.4. Prospects for the Floricultural Industry

The increasing cost of fertilizer inputs is just one reason why growers of horticultural crops in controlled greenhouse growing environments are interested in updated application rates and practices. Across Canada, the horticultural sector, especially greenhouse growers, comes under scrutiny as a possible source of contamination when unacceptable levels of nutrients pollute local waterways [50]. Thus, environmental regulations are becoming increasingly stringent to control the quality of irrigation run-off water [5,71].

Closed subirrigation has been developed for recycling water and nutrients, thereby minimizing fertilizer usage and environmental risks. However, it is still possible to optimize fertilizer use by understanding how nutrients are absorbed and redistributed in plants to meet the needs of the developing flowers. Our modified nutrient delivery strategy combined the removal of the entire nutrient suite at the beginning of reproductive growth with optimizing the nutrient supply during vegetative growth. In all cases, the delivery of both macronutrients and micronutrients using subirrigation could be reduced by at least 75% compared to standard fertilizer formulations, leading to cost savings [72], as well as fewer nutrient-rich solutions to manage, thereby decreasing environmental risks. The validation of these findings using an optimized nutrient solution and modern chrysanthemum cultivars is currently underway in both research and commercial settings.

The next application of this strategy could be drip-irrigated chrysanthemums, which would reduce the requirement for overirrigation to leach salts from the potting medium [73]. Finally, applying our low-input nutrient delivery strategy to other floricultural crops may be possible. Over 5.6 million chrysanthemums and 233 million potted ornamental plants were grown indoors in Canada in 2021 [74]. Our research could improve the overall sustainability of the floricultural industry.

## 4. Materials and Methods

### 4.1. Plant Growth Conditions

The cultivation and growth conditions for *Chrysanthemum morifolium* Ramat. (‘Milton Dark Pink,’ ‘Williamsburg Purple,’ and ‘Mount Aubisque Purple’) have been published previously [3,4,14]. Briefly, the commercial grower (Kuyvenhoeven Greenhouses Inc., Halton Hills, ON, USA) inserted individual unrooted cuttings into Peat Jiffy Plugs amended with 30% minerals (Model CF Hort. Plug 343040-26, Jiffy Products (N.B.) Ltd., Shippagan, NB, Canada), and maintained them for 21 d in the vegetative state for long days in a naturally lit greenhouse (43.581° N, 79.931° W). The rooted cuttings were transported to the University of Guelph (43.314° N, 80.134° W) on the first day of the experiment and individually transplanted into 10-cm-diameter round pots (0.42 L) filled with uncharged soil (BM6 50P No Fert 6600209; Berger, Boisbriand, QC, Canada), which was a mixture of peat moss and perlite (50:50 by volume, pH 5.70–6.15). Background levels of B and Mo in saturated medium extracts of the final peat mixture were 4.6 nmol L^−1^ B and <0.2 nmol L^−1^ Mo, respectively. The potted plants were spaced evenly on four benches, for a total of 16 troughs, in a naturally lit greenhouse maintained at 25 °C and 50% relative humidity day and night.

The plants were organized in a side-by-side split-plot randomized complete block design with four blocks (Figure S2). Each treatment appeared once in each block, and there were 10 plants per treatment. Nutrient treatments served as the main plot and two cultivars served as split-plots. One row of plants around each bench served as a border row and was omitted from the analyses. Four experiments were conducted in total: summer 2021 B (experiment 1; 1 June–20 August) and Mo (experiment 1; 6 July–29 September); and winter/spring 2022 B (experiment 2; 1 February–21 April) and Mo (experiment 2; 15 February–10 May). Three experiments used ‘Milton Dark Pink’ and ‘Williamsburg Purple’ cultivars, whereas Mo experiment 2 used ‘Milton Dark Pink’ and ‘Mount Aubisque Purple’. The B treatments contained 5.00, 2.50, or 1.25 μmol L^−1^ B in experiment 1 and 1.250, 0.625, or 0.313 μmol L^−1^ B in experiment 2 in an otherwise balanced nutrient solution prepared with deionized water (Table S16). The Mo treatments contained 0.500, 0.250, or 0.125 μmol L^−1^ Mo in experiment 1 and 0.125, 0.063, or 0.031 μmol L^−1^ Mo in experiment 2 (Table S17). The composition of the nutrient solutions was essentially as described earlier (Tables S16 and S17) [13].

In summer 2021 (1 June–29 September) experiments, the plants were exposed to long day conditions (12 h light:12 h dark cycle) for 1 week, and vegetative growth was maintained by implementing a night break from 0030 to 0230 h with low-intensity supplemental LED lighting. Then, the plants were pinched and exposed to short days (10 h light:14 h dark cycle) to induce flowering. Conditions were similar in winter/spring 2022 (1 February–10 May) experiments, except that the plants in this experiment remained in long days for an extra 7 d to ensure adequate root development, were pinched 14 d after transplanting, and were provided with low-intensity supplemental LED 94 lighting during the day. Nutrient solutions were supplied to all plants during vegetative growth via a computer-controlled, closed ebb-and-flow subirrigation system every 1–4 d at 1000 h for 5 min to create a 2–3 cm deep flow, which was recycled. At the onset of the reproductive growth (i.e., bud break), all nutrient solutions were replaced with deionized water.

### 4.2. Integrated Pest Management

During the summer of 2021, a sulphur pot was activated to control a small outbreak of powdery mildew. In addition, three biocontrols (Swirskii-system, Degenerans-System, and Aphidius-Mix-System, Biobest Canda Ltd., Leamington, ON, Canada) were dispersed weekly onto each plant, Beleaf^TM^ 50SG Insecticide (ISK Biosciences Corporation, Concord, OH, USA) was applied thrice over the season for thrips, and Avid^®^ 1.9% Miticide/Insecticide (Syngenta Canada Inc., Guelph, ON, Canada) and Forbid^TM^ 240 SC Insecticide/Miticide (Bayer CropScience Inc., Calgary, AB, Canada) were each applied once for spider mites. If present, spider mite webs were manually removed every 2 d. During the winter/spring 2022 experiments, two biocontrols (Swirskii-system and Degenerans-System, Biobest Canada Ltd., Leamington, ON, Canada) were dispersed weekly onto each plant. In addition, Velifer^®^ Biological Insecticide (BASF, Research Triangle Park, NC, USA) was applied once to the thrips.

### 4.3. Data Collection

Total bud/inflorescence number and development (scale from 1 to 6) were determined on a weekly basis for each experimental plant from bud break to harvest (Figure S3; [16]). For the Mo experiments, the leaf chlorophyll content of three recently matured leaves on each plant at bud break was estimated as greenness using a SPAD 502DL Plus Chlorophyll Meter (Konica Minolta, Inc., Tokyo, Japan [75]. At harvest, shoot height from the soil surface to the top of the canopy, shoot fresh mass (FM), and bud/inflorescence FM were measured. Bloom diameter was measured for all opened flowers. The stems/leaves and buds/inflorescences from each plant were dried separately at 95 °C for at least 3 d. From these data, the bud/inflorescence and shoot (including bud/inflorescence) dry mass (DM) were determined for each plant.

The total nutrient composition of the diagnostic leaves was determined at bud break by pooling the ground dried tissues of recently matured leaves from 10 individual plants of each treatment/cultivar replicate and conducting a single analytical determination of a subsample as described previously [17]; the data are reported as the mean of four treatment replicates. The total nutrient composition of the entire shoot (i.e., leaves, stems, inflorescences, and buds) was determined at the final harvest by pooling the dried ground tissues of all plants within each treatment/cultivar replicate and conducting a single analytical determination of the two subsamples. The percent recovery of dried shoot material was used to calculate the total shoot accumulation of any nutrient (*N*_t_) and three indices of use efficiency: nutrient use efficiency (*N_t_*UE = mg shoot DM/mg shoot *N*_t_ content); nutrient utilization efficiency (*N_t_*UtE = mg inflorescence DM/mg shoot *N*_t_ content); nutrient uptake efficiency (*N*_t_UpE = mg shoot *N*_t_ content/mmol L^−1^ *N*_t_ supply (macronutrients in g/mmol L^−1^; micronutrients in mg/μmol L^−1^)) [76].

### 4.4. Statistical Analysis

All data were analyzed using SAS Studio (SAS Institute Inc., Cary, NC, USA), using the PROC GLIMMIX method (α = 0.05). Normality and homogeneity of variance were confirmed before further statistical analyses were performed. Cultivars were initially analyzed together to compare responses to the main effect, and then individually for comparison of the main effect. Data comparisons across time were executed using repeated measure analysis (i.e., inflorescence development and bud/inflorescence number) using a compound symmetry covariance structure. The variance was separated into fixed effects (treatment and cultivar), random effects (block), and all relevant interactions within and between the fixed and random effects. The analyses of variance (ANOVA) were performed, and when effects were significant (*p* ≤ 0.05), the means were compared to each other using Tukey’s honest significant difference test using the slice function.

## Figures and Tables

**Table 1:** Morphological characteristics of two chrysanthemum cultivars supplied with varying levels of B up to bud break in summer 2021 (experiment 1) and winter/spring 2022 (experiment 2).

Cultivar	B Supply (μmol L^−1^)	Shoot			Bud/Inflorescence			
		Height (cm plant^−1^)	DM (g plant^−1^)		DM (g plant^−1^)	Bloom Dia. (cm plant^−1^)	Development (Stage)	Number (Total plant^−1^)
								
Experiment 1								
‘Milton Dark Pink’	5.00	33.79	5.44		1.99	5.86	3.9	34.1*a*
	2.50	35.22	5.59		2.00	5.95	3.9	32.4*ab*
	1.25	34.79	5.21		1.87	5.91	4.0	31.4*b*
								
‘Williamsburg Purple’	5.00	35.26	6.47		2.92	4.56	3.3	36.1
	2.50	34.38	6.39		2.83	4.58	3.4	34.8
	1.25	35.74	6.10		2.58	4.60	3.3	31.7
								
Experiment 2								
‘Milton Dark Pink’	1.25	23.3	2.98*b*		1.30*b*	6.39	4.1	25.6
	0.63	23.1	3.00*b*		1.33*b*	6.44	4.1	25.4
	0.31	24.4	3.35*a*		1.47*a*	6.42	4.3	26.3
								
‘Williamsburg Purple’	1.25	23.6	4.20		2.04	5.48	3.5	23.8
	0.63	23.6	4.28		2.06	5.55	3.5	24.3
	0.31	22.5	3.78		1.78	5.51	3.4	22.6

Means (n = 4) that are significantly different (*p* ≤ 0.05) within columns and cultivars according to Tukey’s honest significant difference test are designated by different letters. Abbreviations: dia., diameter; DM, dry mass.

**Table 2:** Leaf macronutrient composition of two chrysanthemum cultivars supplied with varying levels of B until bud break in summer 2021 (experiment 1) and winter/spring 2022 (experiment 2).

Cultivar	B Supply (μmol L^−1^)	Leaf Concentration (% DM)					
		N	P	K	Ca	Mg	S
							
Experiment 1							
‘Milton Dark Pink’	5.00	5.32	0.91	5.49	1.60	0.55	0.29
	2.50	5.33	0.91	5.30	1.64	0.56	0.29
	1.25	5.26	0.86	5.35	1.50	0.52	0.30
							
‘Williamsburg Purple’	5.00	5.55	0.76	5.54	1.26	0.42	0.30
	2.50	5.63	0.76	5.35	1.27	0.42	0.30
	1.25	5.67	0.78	5.43	1.28	0.42	0.31
							
Experiment 2							
‘Milton Dark Pink’	1.25	6.29	1.01	6.43*b*	1.70	0.70	0.30
	0.63	6.44	1.02	6.60*ab*	1.69	0.74	0.30
	0.31	6.51	1.02	6.95*a*	1.71	0.73	0.29
							
‘Williamsburg Purple’	1.25	6.45	0.92	6.51	1.26	0.50	0.27
	0.63	6.52	0.90	6.50	1.26	0.49	0.28
	0.31	6.60	0.92	6.58	1.28	0.51	0.28

Means (n = 4) that are significantly different (*p* ≤ 0.05) within columns and cultivars according to Tukey’s honest significant difference test are designated by different letters. Each replicate is based on a single analytical determination of a subsample taken from the pooled tissues of 10 individual plants.

**Table 3:** Leaf micronutrient composition of two chrysanthemum cultivars supplied with varying levels of B until flower bud break in summer 2021 (experiment 1) and winter/spring 2022 (experiment 2).

Cultivar	B Supply (μmol L^−1^)	Leaf Concentration (mg kg^−1^ DM)					
		B	Mo	Cu	Zn	Mn	Fe
							
Experiment 1							
‘Milton Dark Pink’	5.00	48.8	2.3	6.4	49.5	85.0	119.8
	2.50	49.0	2.4	7.1	53.5	81.3	116.3
	1.25	46.5	2.4	5.7	42.8	74.0	94.5
							
‘Williamsburg Purple’	5.00	42.0	4.6	5.6	35.3	66.5	91.0
	2.50	42.3	4.8	6.5	44.5	76.0	102.5
	1.25	41.8	4.1	5.2	34.5	79.3	115.3
							
Experiment 2							
‘Milton Dark Pink’	1.25	38.5*a*	2.2	4.6	49.3	66.5	111.0
	0.63	19.3*b*	1.9	4.3	46.3	68.8	98.5
	0.31	11.3*c*	2.0	4.3	43.0	71.8	109.5
							
‘Williamsburg Purple’	1.25	33.8*a*	4.3	3.0	30.3	101.8	102.5
	0.63	19.5*b*	4.2	2.9	28.5	110.5	104.0
	0.31	11.3*c*	3.6	3.0	30.0	115.0	98.5

Means (n = 4) that are significantly different (*p* ≤ 0.05) within columns and cultivars according to Tukey’s honest significant difference test are designated by different letters. Each replicate is based on a single analytical determination of a subsample taken from the pooled tissues of 10 individual plants.

**Table 4:** Morphological characteristics of three chrysanthemum cultivars at harvest and leaf greenness at bud break supplied with varying levels of Mo up to bud break in summer 2021 (experiment 1) and winter/spring 2022 (experiment 2).

Cultivar		Mo Supply (μmol L^−1^)	Shoot				Bud/Inflorescence			
			Height (cm plant^−1^)	DM (g plant^−1^)	SPAD value		DM (g plant^−1^)	Bloom Dia. (cm plant^−1^)	Development (Stage)	Number (Total plant^−1^)
									
	Experiment 1									
‘Milton Dark Pink’		0.500	30.64	4.83	44.02*ab*		1.74*a*	5.58	3.9*a*	35.5
		0.250	32.36	5.69	44.74*a*		2.17*b*	5.72	4.2*b*	36.8
		0.125	30.34	5.05	43.60*b*		1.86*ab*	5.47	3.9*a*	36.5
									
‘Williamsburg Purple’		0.500	30.77	6.61	45.92		2.46	4.53	3.3	33.4
		0.250	30.47	6.56	46.39		2.44	4.53	3.2	33.9
		0.125	32.60	6.75	47.10		2.48	4.64	3.3	32.6
									
	Experiment 2									
‘Milton Dark Pink’		0.125	29.71	4.96	43.16		1.99	6.64	4.2	31.0
		0.063	30.22	4.95	43.70		1.92	6.72	4.1	29.5
		0.031	29.61	4.92	43.13		1.99	6.82	4.3	29.7
									
‘Mount Aubisque Purple’		0.125	30.67	5.80	40.52*a*		2.56	6.45	4.3	23.9
		0.063	30.02	5.83	41.50*b*		2.56	6.43	4.2	24.4
		0.031	29.41	6.08	40.31*a*		2.75	6.39	4.3	24.6

Means (n = 4) that are significantly different (*p* ≤ 0.05) within columns and cultivars according to Tukey’s honest significant difference test are designated by different letters. Each replicate consists of 10 individual plants. SPAD value for each plant is the average of measurements from three recently matured leaves of 10 individual plants per treatment in both experiments. Abbreviation: dia., diameter; DM, dry mass.

**Table 5:** Leaf macronutrient composition of three chrysanthemum cultivars supplied with varying levels of Mo until flower bud break in summer 2021 (experiment 1) and winter/spring 2022 (experiment 2).

Cultivar	Mo Supply (μmol L^−1^)	Leaf Concentration (% DM)					
		N	P	K	Ca	Mg	S
							
Experiment 1							
‘Milton Dark Pink’	0.500	5.03	0.66	5.50	1.40	0.55	0.30
	0.250	4.83	0.57	5.38	1.34	0.52	0.30
	0.125	4.82	0.63	5.34	1.31	0.51	0.29
							
‘Williamsburg Purple’	0.500	4.87	0.54	4.89	1.09	0.35	0.27
	0.250	4.97	0.59	4.98	1.12	0.36	0.28
	0.125	5.09	0.60	5.11	1.12	0.37	0.28
							
Experiment 2							
‘Milton Dark Pink’	0.125	5.08	0.73	5.58	1.19	0.44	0.43
	0.063	5.17	0.69	5.65	1.16	0.42	0.42
	0.031	5.12	0.72	5.53	1.19	0.45	0.42
							
‘Mount Aubisque Purple’	0.125	4.92	0.72*a*	5.99	1.03	0.40	0.50
	0.063	5.09	0.65*b*	6.10	1.02	0.40	0.49
	0.031	5.02	0.69*ab*	6.00	1.04	0.42	0.48

Means (n = 4) that are significantly different (*p* ≤ 0.05) within columns and cultivars according to Tukey’s honest significant difference test are designated by different letters. Each replicate is based on a single analytical determination of a subsample taken from the pooled tissues of 10 individual plants.

**Table 6:** Leaf micronutrient composition of three chrysanthemum cultivars supplied with varying levels of Mo until flower bud break in summer 2021 (experiment 1) and winter/spring 2022 (experiment 2).

Cultivar	Mo Supply (μmol L^−1^)	Leaf Concentration (mg kg^−1^ DM)					
		Mo	B	Cu	Zn	Mn	Fe
							
Experiment 1							
‘Milton Dark Pink’	0.500	1.4	64.8	3.4	23.3	75.3	70.8
	0.250	1.2	63.5	3.2	27.5	66.3	70.3
	0.125	1.5	62.5	3.2	23.3	66.5	82.0
							
‘Williamsburg Purple’	0.500	3.7	48.8	2.7	21.3	60.5	75.3
	0.250	3.4	50.3	2.9	23.5	58.0	74.8
	0.125	2.9	48.8	3.0	21.0	57.3	73.5
							
Experiment 2							
‘Milton Dark Pink’	0.125	1.6*a*	72.5*a*	3.8	34.1	81.3	87.1
	0.063	1.3*b*	71.3*ab*	4.3	33.6	78.9	81.0 *
	0.031	1.1*c*	70.6*b*	4.3	34.1	73.6	83.1 *
							
‘Mount Aubisque Purple’	0.125	1.6*a*	70.8*a*	3.5	28.0	74.5	84.5
	0.063	1.1*b*	70.5*a*	3.5	29.3	79.3	80.8
	0.031	1.0*b*	69.0*b*	3.5	29.0	77.8	84.0

Means (n = 4) that are significantly different (*p* ≤ 0.05) within columns and cultivars according to Tukey’s honest significant difference test are designated by different letters. Each replicate is based on a single analytical determination of a subsample taken from the pooled tissues of 10 individual plants. *, indicates that means are based on two replicates only due to a processing error by the third-party laboratory.

## Data Availability

Not applicable.

## Supplementary Materials

The following supporting information can be downloaded at: https://www.mdpi.com/article/10.3390/plants12122348/s1, Table S1: Comparison of the nutrient solutions used in this study with four commercial fertilizers used in greenhouse floriculture [9,10,11,12]]; Table S2: Summary of significant effects on the morphological characteristics of ‘Milton Dark Pink’ and ‘Williamsburg Purple’ chrysanthemum cultivars supplied with varying levels of B up to bud break in summer 2021 (experiment 1) and winter/spring 2022 (experiment 2); Table S3: Summary of significant effects on the inflorescence development of ‘Milton Dark Pink’ and ‘Williamsburg Purple’ cultivars supplied with varying levels of B up to bud break in summer 2021 (experiment 1) and winter/spring 2022 (experiment 2); Table S4: Summary of significant effects on leaf nutrient composition of ‘Milton Dark Pink’ and ‘Williamsburg Purple’ chrysanthemum cultivars supplied with varying levels of B up to bud break in summer 2021 (experiment 1) and winter/spring 2022 (experiment 2); Table S5: Bud/inflorescence number of two chrysanthemum cultivars supplied with varying levels of B up to bud break in summer 2021 (experiment 1) and winter/spring 2022 (experiment 2); Table S6: Stage of bud/inflorescence development in two chrysanthemum cultivars supplied with varying levels of B up to bud break in summer 2021 (experiment 1) and winter/spring 2022 (experiment 2); Table S7: Macronutrient accumulation by the shoot at harvest of two chrysanthemum cultivars supplied with varying levels of B until bud break in summer 2021 (experiment 1) and winter/spring 2022 (experiment 2); Table S8: Micronutrient accumulation by the shoot at harvest of two chrysanthemum cultivars supplied with varying levels of B until flower bud break in summer 2021 (experiment 1) and winter/spring 2022 (experiment 2); Table S9: Summary of significant effects on the morphological characteristics of ‘Milton Dark Pink’, ‘Williamsburg Purple’, and ‘Mount Aubisque Purple’ chrysanthemum cultivars supplied with varying levels of Mo up to bud break in summer 2021 (experiment 1) and winter/spring 2022 (experiment 2); Table S10: Summary of significant effects on the inflorescence development of ‘Milton Dark Pink’, ‘Williamsburg Purple’, and ‘Mount Aubisque Purple’ chrysanthemum cultivars supplied with varying levels of Mo up to bud break in summer 2021 (experiment 1) and winter/spring 2022 (experiment 2); Table S11: Summary of significant effects on leaf nutrient composition of ‘Milton Dark Pink’, ‘Williamsburg Purple’, and ‘Mount Aubisque Purple’ chrysanthemum cultivars supplied with varying levels of Mo up to bud break in summer 2021 (experiment 1) and winter/spring 2022 (experiment 2); Table S12: Bud/inflorescence number in two chrysanthemum cultivars supplied with varying levels of Mo up to bud break in summer 2021 (experiment 1) and winter/spring 2022 (experiment 2); Table S13: Bud/inflorescence stage of three chrysanthemum cultivars supplied with varying levels of Mo up to bud break in summer 2021 (experiment 1) and winter/spring 2022 (experiment 2); Table S14: Macronutrient accumulation by the shoot at harvest of three chrysanthemum cultivars supplied with varying levels of Mo until flower bud break in summer 2021 (experiment 1) and winter/spring 2022 (experiment 2); Table S15: Micronutrient accumulation by the shoot at harvest of three chrysanthemum cultivars supplied with varying levels of Mo until flower bud break in summer 2021 (experiment 1) and winter/spring 2022 (experiment 2); Table S16: Composition of nutrient solutions used for the two B experiments; Table S17: Composition of nutrient solutions used for the two Mo experiments; Figure S1: Representative plants at harvest at harvest in two chrysanthemum cultivars supplied with varying levels of Mo prior to bud emergence (experiment 2); Figure S2: Example of the split-plot randomized complete block design experimental setup for all experiments; Figure S3: Inflorescence development stages of ‘Milton Dark Pink’ (a), ‘Williamsburg Purple (b), and ‘Mount Aubisque Purple’ (c) chrysanthemums [16].
