# Supplementary material for: Improving Boron and Molybdenum Use Efficiencies in Contrasting Cultivars of Subirrigated Greenhouse-Grown Pot Chrysanthemums
Source: Plants (Basel). 2023 Jun 16;12(12):2348. doi: 10.3390/plants12122348 (PMC10303127; doi:10.3390/plants12122348)
Supplement: Supplementary file 1 [file plants-12-02348-s001.zip › plants-2414514-supplementary.pdf]

Improving Boron and Molybdenum Use Efficiencies in Contrasting Cultivars of Subirrigated Greenhouse-Grown Pot Chrysanthemums

Katherine R. Teeter-Wood 1, Edward J. Flaherty 1, Alyna J. Donetz 1, Gordon J. Hoover 1, William N. MacDonald 2, Da-vid J. Wolyn 1 and Barry J. Shelp 1,*

1 Department of Plant Agriculture, University of Guelph, Guelph, ON N1G 2W1, Canada; ktee-te02@uoguelph.ca (K.R.T.-W.); eflahert@uoguelph.ca (E.J.F.); jane.donetz@gmail.com (A.J.D.); ghoover@uoguelph.ca (G.J.H.); dwolyn@uoguelph.ca (D.J.W.)
2 Agricxulture Department, Niagara College Canada, Niagara-on-the-Lake, ON L0S 1J0, Canada; bmacdonald@niagaracollege.ca
* Correspondence: bshelp@uoguelph.ca; Tel.: +519-931-9878

Supplementary Tables

Table S1. Comparison of the nutrient solutions used in this study with four commercial fertilizers used in greenhouse floriculture.

Nutrient	This study	Modified Sonneveld Solution [12]	Fusion Plant Products 17-5-17 [10]	Peter's Professional 17-3-17 [9]	Hoagland Solution [11]
Macronutrients (mmol L^{-1})					
Nitrogen	7.13	18.50	20.40	20.40	10.5
Phosphorus	1.00	2.60	2.85	1.62	1.00
Potassium	3.51	9.10	7.29	7.29	6.00
Calcium	2.60	6.75	1.25	1.67	5.00
Magnesium	0.58	1.50	0.69	0.86	2.00
Sulfur	0.88	2.25	-	-	1.99
Micronutrients (µmol L^{-1})					
Iron	5.28	25.00	30.10	25.60	15.00
Manganese	2.50	5.00	15.10	12.90	9.10
Zinc	3.50	3.50	12.70	10.80	0.76
Copper	0.75	0.75	13.10	4.72	0.31
Boron	5.00 – 0.31	5.00	31.50	31.50	46.25
Molybdenum	0.500 – 0.031	0.50	2.70	1.45	0.11

Table S2. Summary of significant effects on the morphological characteristics of 'Milton Dark Pink' and 'Williamsburg Purple' chrysanthemum cultivars supplied with varying levels of B up to bud break in summer 2021 (experiment 1) and winter/spring 2022 (experiment 2).

**Effect**	**Shoot height (cm plant^{-1})**	**Shoot DM (g plant^{-1})**	**Inflor. DM (g plant^{-1})**	**Bloom dia. (cm plant^{-1})**	**Bud/inflor. no. (total plant^{-1})**	**Inflor. dev. (stage plant^{-1})**
			Experiment 1			
Treatment						
Cultivar	*	*	*	*	*	*
Treatment x cultivar	*					
			Experiment 2			
Treatment						
Cultivar		*	*	*	*	*
Treatment x cultivar	*	*	*		*	*

*, indicates significance ($P \leq 0.05$). Abbreviations: inflor., inflorescences; dia., diameter; dev., development; DM, dry mass; no., number.

Table S3. Summary of significant effects on the inflorescence development of ‘Milton Dark Pink’ and ‘Williamsburg Purple’ cultivars supplied with varying levels of B up to bud break in summer 2021 (experiment 1) and winter/spring 2022 (experiment 2).

Effect	Experiment 1	Experiment 2
Treatment		
Time	*	*
Cultivar	*	*
Time x treatment		
Treatment x cultivar	*	*
Time x cultivar	*	*
Time x treatment x cultivar		

*, indicates significance (P ≤ 0.05).

Table S4. Summary of significant effects on leaf nutrient composition of ‘Milton Dark Pink’ and ‘Williamsburg Purple’ chrysanthemum cultivars supplied with varying levels of B up to bud break in summer 2021 (experiment 1) and winter/spring 2022 (experiment 2).

Effect	Macronutrient concentration (% DM)						Micronutrient concentration (mg kg^{-1} DM)					
	N	P	K	Ca	Mg	S	B	Mo	Cu	Zn	Mn	Fe
					Experiment 1							
Treatment									*			
Cultivar	*	*		*	*	*	*	*		*		
Treatment x cultivar												
					Experiment 2							
Treatment							*					
Cultivar		*		*	*	*		*	*	*	*	
Treatment x cultivar												

*, indicates significance (P ≤ 0.05).

Table S5. Bud/inflorescence number of two chrysanthemum cultivars supplied with varying levels of B up to bud break in summer 2021 (experiment 1) and winter/spring 2022 (experiment 2).

**Cultivar**	**B supply**	**Days from bud emergence**						
	**(µmol L^{-1})**	**0**	**7**	**14**	**21**	**28**	**35**	**42**
				Experiment 1				
‘Milton Dark Pink’	5.00	33.3	35.3	34.0	32.1	32.7	33.9	-
	2.50	31.1	32.5	32.3	30.9	31.5	32.4	-
	1.25	31.0	32.2	31.6	30.5	30.7	30.9	-
‘Williamsburg Purple’	5.00	29.3*Y*	35.8*Z*	37.8*Z*	37.1*Z*	35.4*Z*	34.4*Z*	36.1*Z*
	2.50	32.0*Y*	36.3*Z*	37.4*Z*	35.3*Z*	33.7*Z*	34.6*Z*	34.8*Z*
	1.25	28.7*Y*	32.8*YZ*	34.3*Z*	32.2*Z*	31.4*Z*	31.5*Z*	31.1*Z*
				Experiment 2				
‘Milton Dark Pink’	1.25	26.7	27.4	26.7	26.9	25.5	-	-
	0.63	26.8	26.6	26.8	26.4	25.7	-	-
	0.31	27.5	27.4	27.1	26.8	26.2	-	-
‘Williamsburg Purple’	1.25	25.3*Y*	28.5*Z*	27.3*Z*	26.9*Z*	26.5*Z*	23.0*Y*	-
	0.63	27.7*Y*	30.2*Y*	29.4*Y*	29.0*Y*	28.3*Y*	24.3*Z*	-
	0.31	25.7*YZ*	28.6*Y*	27.8*Y*	27.2*Y*	26.9*Y*	23.6*Z*	-

Stage was assessed weekly from bud break (stage 1) to harvest. Means (n = 4) within cultivars are not significantly different across treatment according to Tukey’s honest significant difference test ($P \leq 0.05$). Means sharing the same uppercase letters within cultivars are not significantly different across time ($P \leq 0.05$).

Table S6. Stage of bud/inflorescence development in two chrysanthemum cultivars supplied with varying levels of B up to bud break in summer 2021 (experiment 1) and winter/spring 2022 (experiment 2).

**Cultivar**	**B supply (μmol L^{-1})**	**Days from bud emergence**						
		**0**	**7**	**14**	**21**	**28**	**35**	**42**
				Experiment 1				
‘Milton Dark Pink’	5.00	1.0*V*	1.0*V*	1.3*W*	2.2*X*	3.2*aY*	3.9*Z*	-
	2.50	1.0*V*	1.0*V*	1.2*W*	2.1*X*	3.1*abY*	3.9*Z*	-
	1.25	1.0*V*	1.0*V*	1.3*W*	2.2*X*	3.1*bY*	3.9*Z*	-
‘Williamsburg Purple’	5.00	1.0*V*	1.0*V*	1.0*V*	1.4*W*	2.0*X*	2.6*Y*	3.3*Z*
	2.50	1.0*V*	1.0*V*	1.1*V*	1.5*W*	2.0*X*	2.6*Y*	3.4*Z*
	1.25	1.0*V*	1.0*V*	1.0*V*	1.4*W*	1.9*X*	2.5*Y*	3.3*Z*
				Experiment 2				
‘Milton Dark Pink’	1.25	1.0*W*	1.1*W*	1.7*X*	2.8*Y*	4.1*aZ*	-	-
	0.63	1.0*V*	1.2*W*	1.8*X*	2.8*Y*	4.1*abZ*	-	-
	0.31	1.0*V*	1.2*W*	1.8*X*	2.8*Y*	4.3*bZ*	-	-
‘Williamsburg Purple’	1.25	1.0*V*	1.0*V*	1.4*W*	2.0*X*	2.5*Y*	3.6*aZ*	-
	0.63	1.0*V*	1.0*V*	1.4*W*	2.0*X*	2.5*Y*	3.5*aZ*	-
	0.31	1.0*V*	1.0*V*	1.4*W*	2.0*X*	2.5*Y*	3.3*bZ*	-

Stage was assessed weekly from bud break (stage 1) to harvest. Means (n = 4) sharing the same lowercase letters within cultivars are not significantly different across treatment according to Tukey’s honest significant difference test ($P \leq 0.05$). Means sharing the same uppercase letters within cultivars are not significantly different across time ($P \leq 0.05$). Stage 1: a formed bud that is completely closed; stage 2 the bloom beginning to emerge from the bud with visible petal colour; stage 3: the bloom opening with fully vertical petals but still mostly closed with sepals approximately half the petal length; stage 4: the bloom opening and mostly open; stage 5: a fully opened bloom; and stage 6: a fully opened bloom in the early stages of petal-tip senescence with fully opened disk flowers.

Table S7. Macronutrient accumulation by the shoot at harvest of two chrysanthemum cultivars supplied with varying levels of B until bud break in summer 2021 (experiment 1) and winter/spring 2022 (experiment 2).

**Cultivar**	**B supply**	**Shoot accumulation (g shoot^{-1})**					
	**(µmol L^{-1})**	**N**	**P**	**K**	**Ca**	**Mg**	**S**
			Experiment 1				
'Milton Dark Pink'	5.00	1.68	0.32	1.93	0.64	0.22	1.10
	2.50	1.74	0.33	2.01	0.67	0.23	1.13
	1.25	1.62	0.31	1.86	0.61	0.21	1.03
'Williamsburg Purple'	5.00	1.96	0.33	2.29	0.59	0.21	1.19
	2.50	1.97	0.33	2.28	0.58	0.21	1.14
	1.25	1.92	0.32	2.23	0.57	0.21	1.13
			Experiment 2				
'Milton Dark Pink'	1.25	1.11	0.20	1.22	0.36	0.15	0.67
	0.63	1.07	0.20	1.18	0.35	0.14	0.67
	0.31	1.12	0.21	1.33	0.38	0.15	0.78
'Williamsburg Purple'	1.25	1.20	0.20	1.33	0.31	0.12	0.76
	0.63	1.23	0.21	1.39	0.33	0.13	0.80
	0.31	1.19	0.19	1.31	0.30	0.12	0.72

Each replicate is based on a single analytical determination of a subsample taken from the pooled tissues of 10 individual plants. Means (n = 4) within columns and cultivars are not significantly different according to the Tukey's honest significant difference test ($P \leq 0.05$).

Table S8. Micronutrient accumulation by the shoot at harvest of two chrysanthemum cultivars supplied with varying levels of B until flower bud break in summer 2021 (experiment 1) and winter/spring 2022 (experiment 2).

**Cultivar**	**B supply**	**Shoot accumulation (mg shoot^{-1})**					
	**(µmol L^{-1})**	**B**	**Mo**	**Cu**	**Zn**	**Mn**	**Fe**
			Experiment 1				
‘Milton Dark Pink’	5.00	2.26*a*	0.23	0.30*ab*	2.22*ab*	3.75	5.28
	2.50	1.67*b*	0.24	0.32*a*	2.39*a*	3.77	6.78
	1.25	1.19*c*	0.22	0.28*b*	2.10*b*	3.36	4.10
‘Williamsburg Purple’	5.00	2.33*a*	0.24	0.36	2.31	3.42	4.93
	2.50	1.88*a*	0.24	0.37	2.50	3.72	5.09
	1.25	1.36*b*	0.23	0.34	2.20	3.72	5.47
			Experiment 2				
‘Milton Dark Pink’	1.25	0.52	0.15	0.12	1.04	2.01	2.14
	0.63	0.48	0.15	0.11	1.02	1.44	1.79
	0.31	0.58	0.15	0.12	1.16	1.73	2.22
‘Williamsburg Purple’	1.25	0.52	0.15	0.12	1.01	2.25	2.37
	0.63	0.54	0.16	0.12	1.06	2.58	2.73
	0.31	0.46	0.16	0.11	0.93	2.50	2.29

Each replicate is based on a single analytical determination of a subsample taken from the pooled tissues of 10 individual plants. Means (n = 4) that are significantly different ($P \leq 0.05$) within columns and cultivars according to the Tukey’s honest significant difference test are designated by different letters.

Table S9. Summary of significant effects on the morphological characteristics of 'Milton Dark Pink', 'Williamsburg Purple', and 'Mount Aubisque Purple' chrysanthemum cultivars supplied with varying levels of Mo up to bud break in summer 2021 (experiment 1) and winter/spring 2022 (experiment 2).

Effect	Shoot height (cm plant^{-1})	Shoot DM (g plant^{-1})	Inflor. DM (g plant^{-1})	Bloom dia. (cm plant^{-1})	Bud/inflor. no. (total plant^{-1})	Inflor. dev. (stage plant^{-1})	Greenness (SPAD value)
				Experiment 1			
Treatment							
Cultivar		*	*	*	*	*	*
Treatment x cultivar	*	*	*	*		*	*
				Experiment 2			
Treatment							*
Cultivar		*	*	*	*	*	*
Treatment x cultivar				*			

*, indicates significance ($P \leq 0.05$) Greenness was determined at bud emergence. Abbreviations: inflor., inflorescence; dia., diameter; dev., development; DM, dry mass.

Table S10. Summary of significant effects on the inflorescence development of ‘Milton Dark Pink’, ‘Williamsburg Purple’, and ‘Mount Aubisque Purple’ chrysanthemum cultivars supplied with varying levels of Mo up to bud break in summer 2021 (experiment 1) and winter/spring 2022 (experiment 2).

Effect	Experiment 1	Experiment 2
Treatment		
Time	*	*
Cultivar	*	*
Time x treatment	*	
Treatment x cultivar	*	*
Time x cultivar	*	*
Time x treatment x cultivar	*	

*, indicates significance ($P \leq 0.05$).

Table S11. Summary of significant effects on leaf nutrient composition of ‘Milton Dark Pink’, ‘Williamsburg Purple’, and ‘Mount Aubisque Purple’ chrysanthemum cultivars supplied with varying levels of Mo up to bud break in summer 2021 (experiment 1) and winter/spring 2022 (experiment 2).

Effect	Macronutrient concentration (% DM)						Micronutrient concentration (mg kg^{-1} DM)					
	**N**	**P**	**K**	**Ca**	**Mg**	**S**	**Mo**	**B**	**Cu**	**Zn**	**Mn**	**Fe**
						Experiment 1						
Treatment												
Cultivar			*	*	*	*	*	*	*			
Treatment x cultivar												
						Experiment 2						
Treatment							*	*				
Cultivar	*	*	*	*	*	*	*	*	*	*		
Treatment x cultivar												

*, indicates significance ($P \leq 0.05$).

Table S12. Bud/inflorescence number in two chrysanthemum cultivars supplied with varying levels of Mo up to bud break in summer 2021 (experiment 1) and winter/spring 2022 (experiment 2).

**Cultivar**	**Mo supply (μmol L^{-1})**	**Days from bud emergence** 0	7	14	21	28	35	42
		Experiment 1						
'Milton Dark Pink'	0.500	35.7	36.8	35.0	35.1	35.6	35.8	-
	0.250	37.5	39.6	37.0	38.4	37.5	38.3	-
	0.125	37.3	38.0	35.9	35.1	36.2	36.6	-
'Williamsburg Purple'	0.500	34.6	36.4	35.0	33.4	34.2*ab*	34.2*a*	33.1
	0.250	35.1	37.4	34.7	34.7	34.6*bc*	34.0*ab*	34.6
	0.125	34.4	36.5	34.1	34.3	33.5*ac*	32.7*b*	32.6
		Experiment 2						
'Milton Dark Pink'	0.125	32.9	32.2	32.4	32.1	31.0	-	-
	0.063	30.5	30.8	31.1	30.4	29.4	-	-
	0.031	31.1	30.*8*	30.6	30.7	29.7	-	-
'Mount Aubisque Purple'	0.125	25.5	25.5	24.3	25.6	24.7	24.1*ab*	23.9*ab*
	0.063	26.3	26.3	26.0	26.0	25.3	25.1*ab*	24.4*ac*
	0.031	25.6	26.1	25.5	26.6	25.9	24.9*bc*	24.6*bc*

Means (n =4) sharing the same lowercase letters within cultivars are not significantly different across treatment according to Tukey's honest significant difference test ($P \leq 0.05$). Means within cultivars are not significantly different across time ($P \leq 0.05$).

Table S13. Bud/inflorescence stage of three chrysanthemum cultivars supplied with varying levels of Mo up to bud break in summer 2021 (experiment 1) and winter/spring 2022 (experiment 2).

**Cultivar**	**Mo supply (μmol L^{-1})**	**Days from bud emergence** 0	7	14	21	28	35	42
				Experiment 1				
'Milton Dark Pink'	0.500	1.0	1.3	2.1*a*	2.8*a*	3.3*a*	3.9*a*	-
	0.250	1.1	1.5	2.4*b*	3.1*b*	3.6*b*	4.2*b*	-
	0.125	1.0	1.4	2.2*ab*	2.9*ab*	3.3*a*	3.8*ab*	-
'Williamsburg Purple'	0.500	1.0*V*	1.0*V*	1.1*V*	1.6*W*	2.0*X*	2.5*Y*	3.3*Z*
	0.250	1.0*V*	1.0*V*	1.1*V*	1.5*W*	2.0*X*	2.6*Y*	3.2*Z*
	0.125	1.0*V*	1.0*V*	1.1*V*	1.6*W*	2.0*X*	2.6*Y*	3.3*Z*
				Experiment 2				
'Milton Dark Pink'	0.125	1.1*W*	1.2*W*	2.0*X*	3.1*Y*	4.2*Z*	-	-
	0.063	1.1*W*	1.2*W*	2.0*X*	3.0*Y*	4.1*Z*	-	-
	0.031	1.1*W*	1.3*W*	2.1*X*	3.1*Y*	4.3*Z*	-	-
'Mount Aubisque Purple'	0.125	1.0*T*	1.4*U*	2.4*V*	3.0*W*	3.4*X*	3.8*Y*	4.3*Z*
	0.063	1.0*T*	1.4*U*	2.3*V*	3.0*W*	3.3*X*	3.7*Y*	4.3*Z*
	0.031	1.0*T*	1.4*U*	2.2*V*	2.9*W*	3.3*X*	3.7*Y*	4.3*Z*

Stage was assessed weekly from bud break (stage 1) to harvest. Data represent the mean of four treatment replicates rounded to the nearest tenth; each replicate consists of 10 individual plants. Means (n = 4) sharing the same lowercase letters within cultivars are not significantly different across treatment according to Tukey's honest significant difference test ($P \leq 0.05$). Means sharing the same uppercase letters within cultivars are not significantly different across time ($P \leq 0.05$). Stage 1: a formed bud that is completely closed; stage 2 the bloom beginning to emerge from the bud with visible petal colour; stage 3: the bloom opening with fully vertical petals but still mostly closed with sepals approximately half the petal length; stage 4: the bloom opening and mostly open; stage 5: a fully opened bloom; and stage 6: a fully opened bloom in the early stages of petal-tip senescence with fully opened disk flowers.

Table S14. Macronutrient accumulation by the shoot at harvest of three chrysanthemum cultivars supplied with varying levels of Mo until flower bud break in summer 2021 (experiment 1) and winter/spring 2022 (experiment 2).

**Cultivar**	**Mo supply (μmol L^{-1})**	**Shoot accumulation (g $shoot^{-1}$)**					
		**N**	**P**	**K**	**Ca**	**Mg**	**S**
			Experiment 1				
‘Milton Dark Pink’	0.500	1.45	0.26	1.84	0.56*b*	0.18	1.03
	0.250	1.63	0.29	2.08	0.64*a*	0.21	1.17
	0.125	1.49	0.27	1.89	0.58*ab*	0.20	1.05
‘Williamsburg Purple’	0.500	1.67	0.27	2.04	0.51	0.18	1.00
	0.250	1.74	0.28	2.08	0.51	0.18	1.00
	0.125	1.74	0.28	2.11	0.52	0.17	1.04
			Experiment 2				
‘Milton Dark Pink’	0.125	1.37	0.24	1.58	0.52	0.18	0.91
	0.063	1.37	0.24	1.58	0.51	0.18	0.91
	0.031	1.34	0.24	1.57	0.52	0.18	0.94
‘Mount Aubisque Purple’	0.125	1.38	0.24	1.68	0.49	0.16	1.40
	0.063	1.45	0.24	1.71	0.49	0.17	1.37
	0.031	1.50	0.24	1.75	0.50	0.17	1.35

Each replicate is based on a single analytical determination of a subsample taken from the pooled tissues of 10 individual plants. Means (n = 4) that are significantly different ($P \leq 0.05$) within columns and cultivars according to the Tukey’s honest significant difference test are designated by different letters.

Table S15. Micronutrient accumulation by the shoot at harvest of three chrysanthemum cultivars supplied with varying levels of Mo until flower bud break in summer 2021 (experiment 1) and winter/spring 2022 (experiment 2).

**Cultivar**	**Mo supply (μmol L^{-1})**	**Shoot accumulation (mg shoot^{-1})**					
		**Mo**	**B**	**Cu**	**Zn**	**Mn**	**Fe**
		Summer 2021					
‘Milton Dark Pink’	0.500	0.16*a*	2.04*b*	0.49	1.70	2.74	3.06
	0.250	0.14*ab*	2.29*a*	0.46	1.92	3.00	3.46
	0.125	0.08*b*	2.09*ab*	0.57	1.62	2.98	2.93
‘Williamsburg Purple’	0.500	0.21*a*	2.06	0.25	1.63	3.02	3.90
	0.250	0.15*b*	2.14	0.25	1.65	3.09	3.38
	0.125	0.12*b*	2.08	0.24	1.70	2.87	3.74
		Winter/Spring 2022					
‘Milton Dark Pink’	0.125	0.09*a*	2.07	0.16	1.53	3.26*ab*	2.51
	0.063	0.05*b*	2.05	0.17	1.55	3.67*a*	2.47
	0.031	0.05*b*	2.08	0.17	1.46	2.28*b*	2.36
‘Mount Aubisque Purple’	0.125	0.13	2.36	0.21	1.78	3.45	3.30
	0.063	0.08	2.42	0.20	1.76	3.48	3.28
	0.031	0.08	2.48	0.20	1.81	3.68	3.79

Each replicate is based on a single analytical determination of a subsample taken from the pooled tissues of 10 individual plants. Means (n = 4) that are significantly different ($P \leq 0.05$) within columns and cultivars according to the Tukey’s honest significant difference test are designated by different letters.

Table S16. Composition of nutrient solutions used for the two B experiments.

**Macronutrient (mmol L^{-1})**		**Micronutrient (µmol L^{-1})**		**Treatment**		
				**T1**	**T2**	**T3**
$Ca(NO_3)_2$	2.60	Fe-EDDHA	5.28			
KNO_3	1.93	$MnSO_4$	2.50			
KH_2PO_4	1.00	$ZnSO_4$	3.50			
K_2SO_4	0.29	$CuSO_4$	0.75			
$MgSO_4$	0.58	Na_2MoO_4	0.50			
		$Na_2B_4O_7$	Expt. 1	5.00	2.50	1.25
		$Na_2B_4O_7$	Expt. 2	1.25	0.63	0.31

Table S17. Composition of nutrient solutions used for the two Mo experiments.

**Macronutrient (mmol L^{-1})**		**Micronutrient (µmol L^{-1})**		**Treatment**		
				**T1**	**T2**	**T3**
$Ca(NO_3)_2$	2.60	Fe-EDDHA	5.28			
KNO_3	1.93	$MnSO_4$	2.50			
KH_2PO_4	1.00	$ZnSO_4$	3.50			
K_2SO_4	0.29	$CuSO_4$	0.75			
$MgSO_4$	0.58	$Na_2B_4O_7$	5.00			
		Na_2MoO_4	Expt. 1	0.500	0.250	0.125
		Na_2MoO_4	Expt. 2	0.125	0.063	0.031

Supplementary Figures

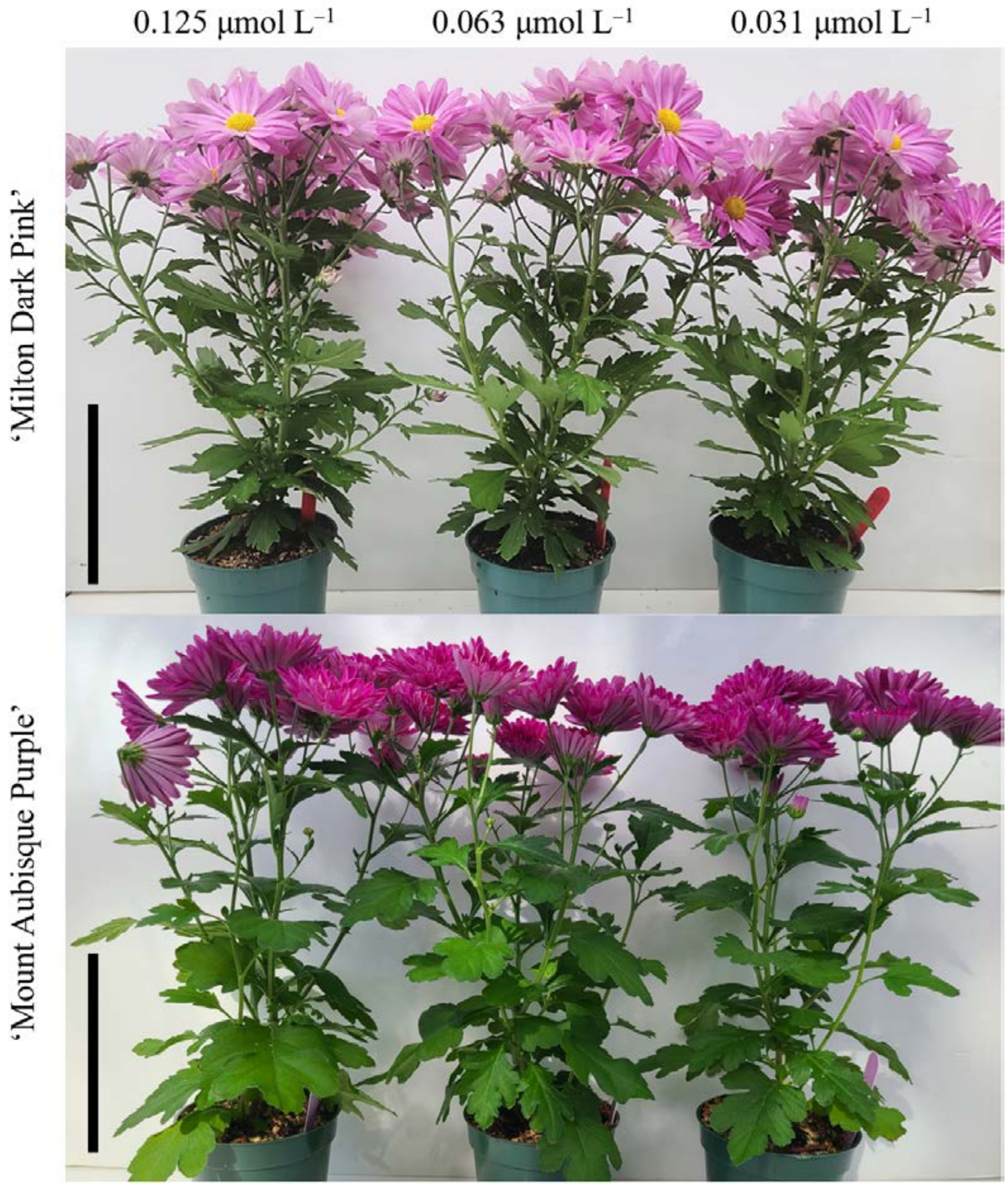

Figure S1. Representative plants at harvest at harvest in two chrysanthemum cultivars supplied with varying levels of Mo prior to bud emergence (experiment 2). Black scale bars = 10 cm.

C1	C1	C1	C2	C2	C2	C1	C1	C1	C1	C1	C2	C2	C1	C1	C2
C1	C1	C1	C2	C2	C2	C1	C1	C1	C1	C1	C2	C2	C1	C1	C2
C1	C1	C1	C2	C2	C2	C1	C1	C1	C1	C1	C2	C2	C1	C1	C2
C1	C1	C1	C2	C2	C2	C1	C1	C1	C1	C1	C2	C2	C1	C1	C2
C1	C1	C1	C2	C2	C2	C1	C1	C1	C1	C1	C2	C2	C1	C1	C2
C1	C1	C1	C2	C2	C2	C1	C1	C1	C1	C1	C2	C2	C1	C1	C2
C1	C1	C1	C2	C2	C2	C1	C1	C1	C1	C1	C2	C2	C1	C1	C2
C1	C1	C1	C2	C2	C2	C1	C1	C1	C1	C1	C2	C2	C1	C1	C2
C1	C1	C1	C2	C2	C2	C1	C1	C1	C1	C1	C2	C2	C1	C1	C2
C1	C1	C1	C2	C2	C2	C1	C1	C1	C1	C1	C2	C2	C1	C1	C2
C1	C1	C1	C2	C2	C2	C1	C1	C1	C1	C1	C2	C2	C1	C1	C2
C2	C2	C2	C1	C1	C1	C2	C2	C2	C2	C2	C1	C1	C2	C2	C1
C2	C2	C2	C1	C1	C1	C2	C2	C2	C2	C2	C1	C1	C2	C2	C1
C2	C2	C2	C1	C1	C1	C2	C2	C2	C2	C2	C1	C1	C2	C2	C1
C2	C2	C2	C1	C1	C1	C2	C2	C2	C2	C2	C1	C1	C2	C2	C1
C2	C2	C2	C1	C1	C1	C2	C2	C2	C2	C2	C1	C1	C2	C2	C1
C2	C2	C2	C1	C1	C1	C2	C2	C2	C2	C2	C1	C1	C2	C2	C1
C2	C2	C2	C1	C1	C1	C2	C2	C2	C2	C2	C1	C1	C2	C2	C1
C2	C2	C2	C1	C1	C1	C2	C2	C2	C2	C2	C1	C1	C2	C2	C1
C2	C2	C2	C1	C1	C1	C2	C2	C2	C2	C2	C1	C1	C2	C2	C1
C2	C2	C2	C1	C1	C1	C2	C2	C2	C2	C2	C1	C1	C2	C2	C1
C2	C2	C2	C1	C1	C1	C2	C2	C2	C2	C2	C1	C1	C2	C2	C1
B	T1	T3	T2	T3	T2	T1	B	B	T1	T2	T3	T1	T3	T2	B
Block 1				Block 2				Block 3				Block 4			
Bench 1								Bench 2							

Figure S2. Example of the split-plot randomized complete block design experimental setup for all experiments*. Each of the four blocks consisted of three experimental troughs, each containing ten experimental plants of both ‘Milton Dark Pink’ (Cultivar 1 = C1) and ‘Williamsburg Purple’ or ‘Mount Aubisque Purple’ (Cultivar = C2). The colour of the troughs indicate treatment: treatment 1 (T1), orange; treatment 2 (T2), red; treatment 3 (T3), yellows. Plants coloured in blue were border (B) plants were not included in the analyses and received treatment 1.

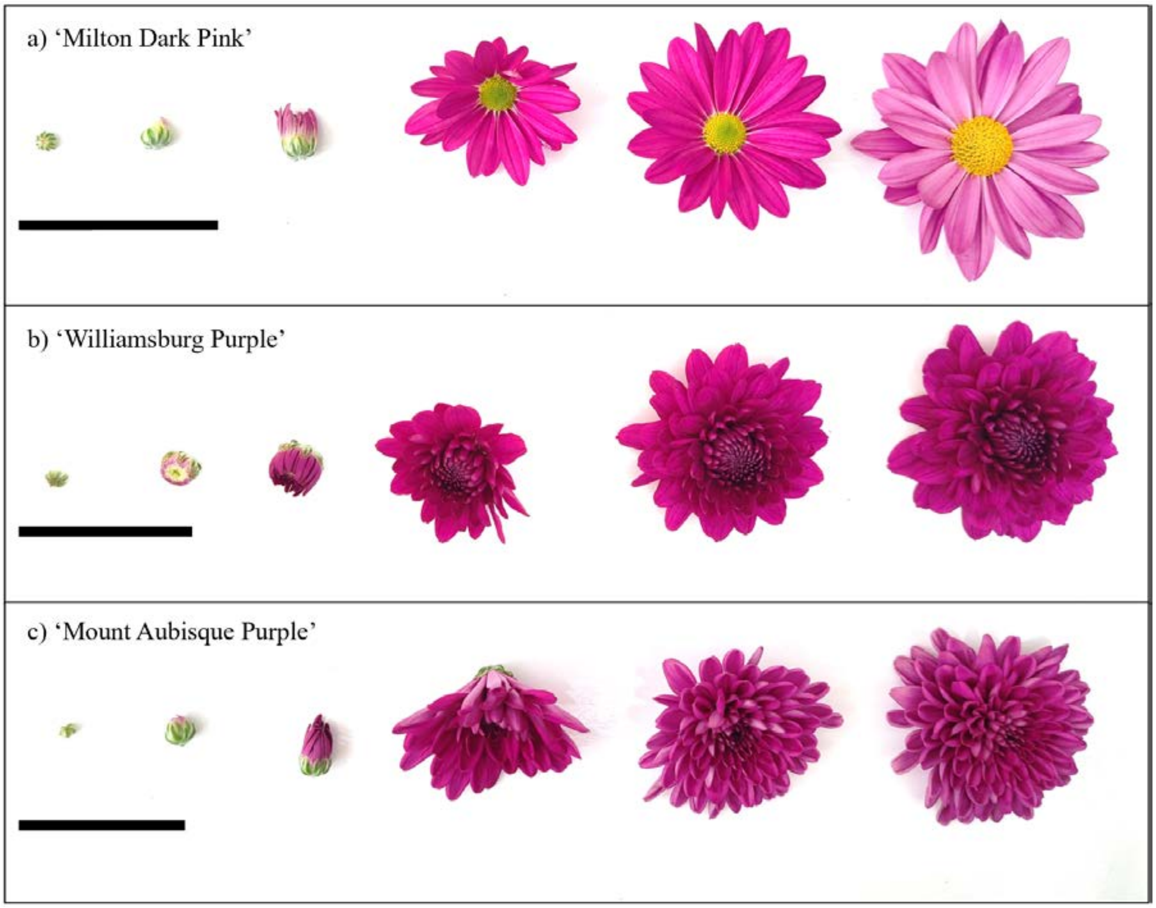

Figure S3. Inflorescence development stages of 'Milton Dark Pink' (a), 'Williamsburg Purple (b), and 'Mount Aubisque Purple' (c) chrysanthemums. Scale from 1-6 from left to right. Stage 1: a formed bud that is completely closed; stage 2 the bloom beginning to emerge from the bud with visible petal colour; stage 3: the bloom opening with fully vertical petals but still mostly closed with sepals approximately half the petal length; stage 4: the bloom opening and mostly open; stage 5: a fully opened bloom; and stage 6: a fully opened bloom in the early stages of petal-tip senescence with fully opened disk flowers. Black scale bar = 5 cm. [16].
